# Diversification of Vascular Occlusions and Crystal Deposits in the Xylem Sap Flow of Five Tunisian Grapevines

**DOI:** 10.3390/plants11162177

**Published:** 2022-08-22

**Authors:** Badra Bouamama-Gzara, Hassene Zemni, Noomene Sleimi, Abdelwahed Ghorbel, Lassaad Gzara, Naima Mahfoudhi

**Affiliations:** 1 Laboratory of Plant Molecular Physiology, Center of Biotechnology of Borj-Cédria, University of Carthage, BP. 905, Hammam-Lif 2050, Tunisia; 2 Laboratory RME—Resources, Materials and Ecosystems, Faculty of Sciences of Bizerte, University of Carthage, Bizerte 7021, Tunisia; 3 Center of Excellence in Desalination Technology, King Abdulaziz University, P.O. Box 80200, Jeddah 21589, Saudi Arabia; 4 Laboratory of Plant Protection, National Institute of Agronomic Research of Tunisia, University of Carthage Rue Hedi Karray, El Menzah 1004, Tunisia

**Keywords:** Energy Dispersive X-ray spectroscopy, xylem sap, environmental scanning electron microscopy, obstruction, vessel elements

## Abstract

Xylem vessels are essential pivotal organs in bulk hydraulic flow through the whole woody plant. However, environmental constraints generate disagreements in xylem structures, which are characterized by air emboli and occlusions formations, compromising water conductivity in grapevines. The aim of this work was to explore xylem morphology dynamics through the xylem sap flow of five Tunisian grapevine cultivars during the natural bleeding sap periods of 2019, 2021, and 2022. In fact, Sakasly, Khamri, Hencha, Razegui1, and Razegui2 rain-fed grapevine cultivars revealed differential responses towards xylem sap movement. The results demonstrated that the xylem sap flow was significantly more abundant in 2019 than 2021 and 2022 bleeding sap campaigns. A variation was revealed between the cultivars regarding the xylem sap flow. In fact, Sakasly gave the best xylem flow during the three campaigns. Razegui1 and Razegui2 registered approximately similar xylem sap flow, while Hencha and Khamri present the lowest sap fluxes during the three campaigns. Moreover, several vascular occlusions forms were identified from stem cross sections using environmental scanning electron microscopy (ESEM), including tyloses, gels, starch, and gum deposits. The highest occlusion number was observed in Sakasly, Razegui1, and Razegui2 cultivars. Among different biogenic calcium shapes, several were observed for the first time in grapevine, including multi-faceted druse, cubic, crystalline sand, styloids, spherical, or drop-like structures. Considering their lower flow and totally blocked vessels, both Hencha and Khamri confirmed their susceptibility to environmental constraints. However, Sakasly, Razegui1, and Razegui2 cultivars presented higher tolerance according to their sap flow and xylem morphology.

## 1. Introduction

Environmental constraints, including water deficit and vascular diseases, have a profound impact on viticulture all around the world [1,2]. Currently, several grapevine cultivars with natural disease resistance have been developed using breeding programs [3,4] and some biotechnological alternatives, such as rhizosphere bioengineering [5,6]. Most of the studies are interested with cultivars and wild accessions which present less susceptibility to pest diseases [7,8]. In fact, grapevine xylem sap, defined as charged water flowing into xylem conduits, is considered as an essential component of the plant nutrition process [9]. Xylem sap also plays a crucial role regarding hydraulic function [10]. The xylem sap is described as a phenomenon indicating an increasing of root activity in early spring, prior to bud break. Its occurrence within the tree guarantees plant growth resumption after dormancy. In perennial plants, such as grapevine, xylem sap exudates are transported unidirectionally all over the tree through the xylem system [11,12]. On the other hand, the salt concentrations in the xylem sap, exert a lower osmotic potential and water diffusion leads to a “root pressure” responsible of the grapevine sap bleeding. During early spring, leaves transpiration increases generating a negative pressure into the xylem and subsequently the sap bleeding is stopped [13]. During environmental disturbances, the water bulk function may be disturbed leading to potential decline in the xylem sap transport [14]. Such constraints create several disorders in xylem structures, which are characterized by air emboli and occlusions formations compromising water conductivity in grapevine [15,16]. Pests, such as endophytic bacteria and fungi, may grow within the bundles and further colonize the plant vascular systems leading to the occlusion of xylem structures [17] and subsequent decrease in hydraulic functionality [14]. To trigger the embolism, the hosted plant synthesizes secondary metabolites, including gum plugs, pectins [18], and other xylem vessel occlusions, such as tyloses and fibrillary networks [19]. In addition, several plant species with ability to discern the embolism can restore the hydraulic functionality by the activation of mechanisms such starch hydrolysis, providing sufficient carbon in parenchyma cells adjacent to xylem vessels [16]. It has also been reported that infected plants develop actively vascular occlusions and become less susceptible to both biotic and abiotic stresses [20].

The specialized cells where crystals are formed are called idioblasts. Crystal idioblasts contain several appearances, number and sizes. The crystal diversity depends on taxa and can be classified in five groups, including prismatic, druses, styloides, crystal sand, and raphide-like shape structures [21,22]. Their presence within plant cells depends on physico-chemical and biological features. Their development can be under a genetic control process [23]. It is essential to recognize grapevine cultivars possessing abilities to overcome environmental constraints. The sap flow and its relationship with the micromorphology of the wood can be considered as good indicators to recognize vulnerable and less vulnerable grapevine cultivars. For this reason, the aims of the present work were to compare xylem sap fluxes of five rain-fed grapevine cultivars during three xylem sap campaigns, as well as to explore the anatomical features of their vessel systems.

## 2. Results

**Figure 1:**
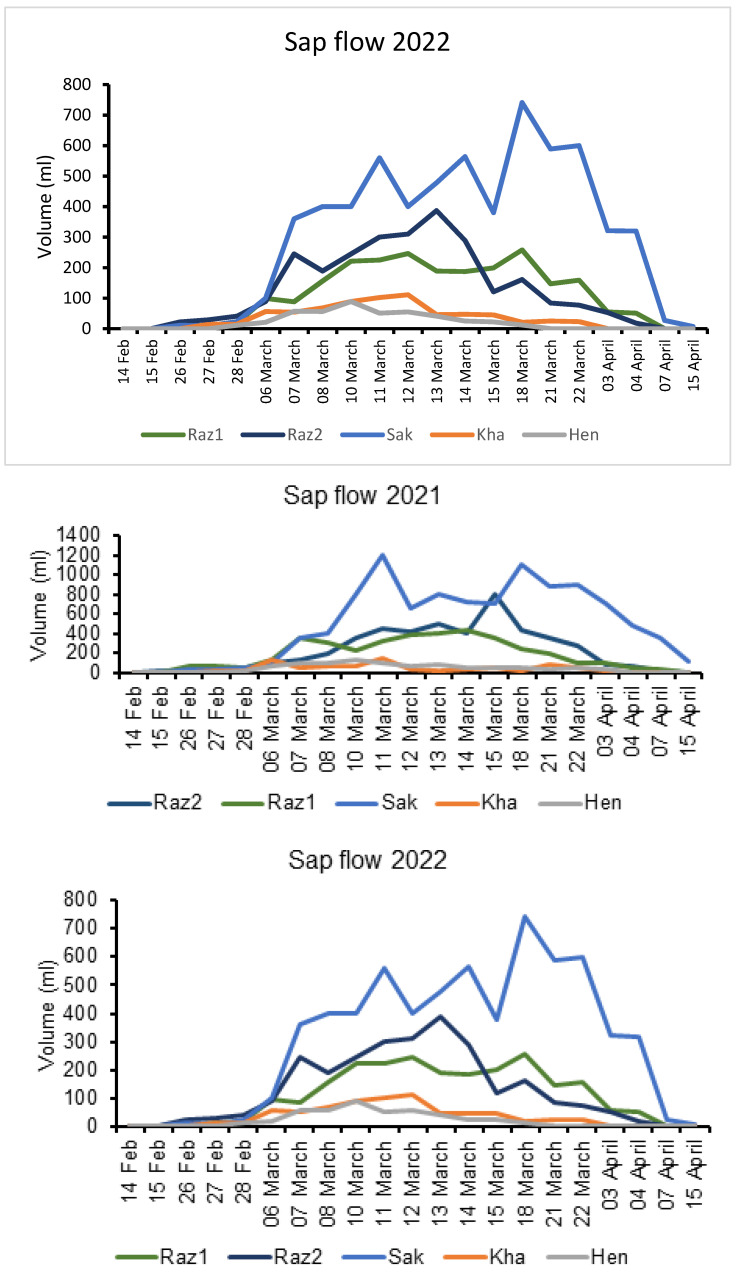
Xylem Sap flow in five Tunisian grapevine cultivars during the bleeding period of 2019, 2021 and 2022. Abbreviations: Sak Sakasly, Hen Hencha, Kha: Khamri, RazI: Razegui1, Raz2: Razegui2.

The emerging sap from pruned woody canes extremities was the first apparent reaction proving the renewal of grapevines metabolic activity after dormancy period. Bleeding period begins in February and may last more than 10 weeks in Northern Tunisian vineyards. We observed that the fluxes were low at the beginning of the three bleeding sap periods (Figure 1). They progressively increased, reaching their maximum just before budburst, which coincided from 4 to 22 March (almost 18 days). The xylem sap volume exuded during 2019, 2021, and 2022 bleeding periods depended on the cultivars.

The maximum of bleeding sap registered for 2019 was 5.471, 4.7 and 3.736 mL/min for Sakasly, Razegui2 and Razegui1, respectively, while the lowest rates were 2.781 and 0.913 mL/min for Hencha and Khamri, respectively. The total xylem sap collected over two months resulted in 11.161 L for Sakasly, 9.59 L and 7.622 L respectively for Razegui1 and Razegui2. For Hencha and Khamri, the collected volumes were 5.675 L and 1.863 L, respectively, for a duration of 17 h per day.

For 2021, the xylem sap detected was 5.084, 2.311 and 1.857 mL/min for Sakasly, Razegui2, and Razegui1, respectively. On the other hand, 0.434 and 0.367 mL/min are the fluxes of Hencha and Khamri respectively. The total xylem sap measured over two months resulted in 10.373 L for Sakasly, 4.714 L and 3.720 L for Razegui2 and Razegui1, respectively. Volumes of 0.887 L and 0.749 L were registered for Hencha and Khamri, respectively.

**Figure 2:**
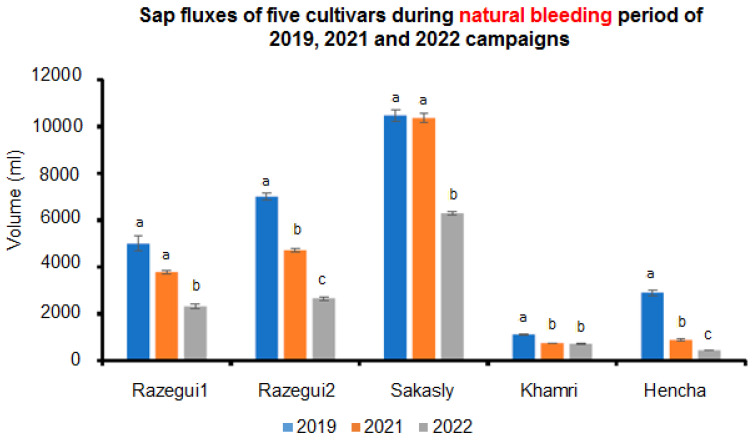
Comparison of the total xylem sap fluxes of five Tunisian grapevine cultivars measured during natural bleeding sap periods of 2019, 2021 and 2022. Different letters indicate significant differences among bleeding sap periods and cultivars and according to a repeated measures ANOVA (*p* ≤ 0.05). Values are means ± SE.

For 2022 bleeding sap period, values of 3.083, 1.304 and 1.132 mL/min were measured respectively for Sakasly, Razegui2, and Razegui1. Meanwhile, 0.351 and 0.215 mL/min were detected for Khamri and Hencha, respectively. The total xylem sap collected for the same period resulted in 6.91 L for Sakasly, 2.66 L for Razegui2 and 2.31 L for Razegui1. For Khamri and Hencha, the collected volumes were 0.716 L and 0.44 L respectively. There was a significant statistical interaction (*p* ≤ 0.05) between cultivars and the year of collect (Figure 2). In fact, the results demonstrated that the xylem sap flow was significantly more abundant in 2019 than in 2021 and 2022 campaigns of collect. On the other hand, Sakasly cultivar presented the biggest fluxes during the three bleeding sap campaigns.

**Figure 3:**
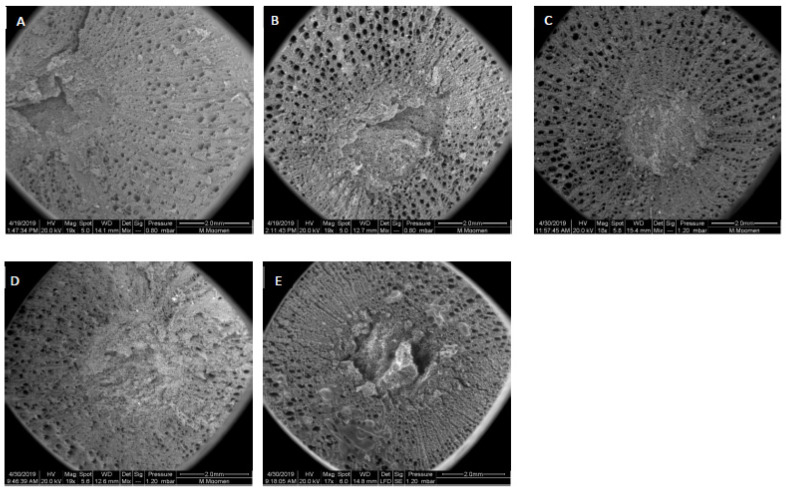
General view and comparison of freshly stem cross sections visualized by Environmental Scanning Electron Microscopy corresponding to five local and rain-fed grapevine (**A**): Hencha, (**B**) Sakasly, (**C**); Razegui2, (**D**); Khamri, (**E**); Razegui1.

The structure of vessel elements was investigated using ESEM analysis in terms of the imbalance found in the flow repartition among the five cultivars. The stem cross sections revealed differences in the vessel arrangement on the cultivars. The spatial distribution of the xylem was diffuse in Hencha and Khamri cultivars (Figure 3A,D). These structures appeared obstructed in various regions, preventing the movement of the sap flow. Moreover, Hencha and Khamri seemed to present more non-functional areas. In contrast, as it was shown in Figure 3B,C,E that Sakasly, Razegui1, and Razegui2, exhibited more functional vessels elements.

**Figure 4:**
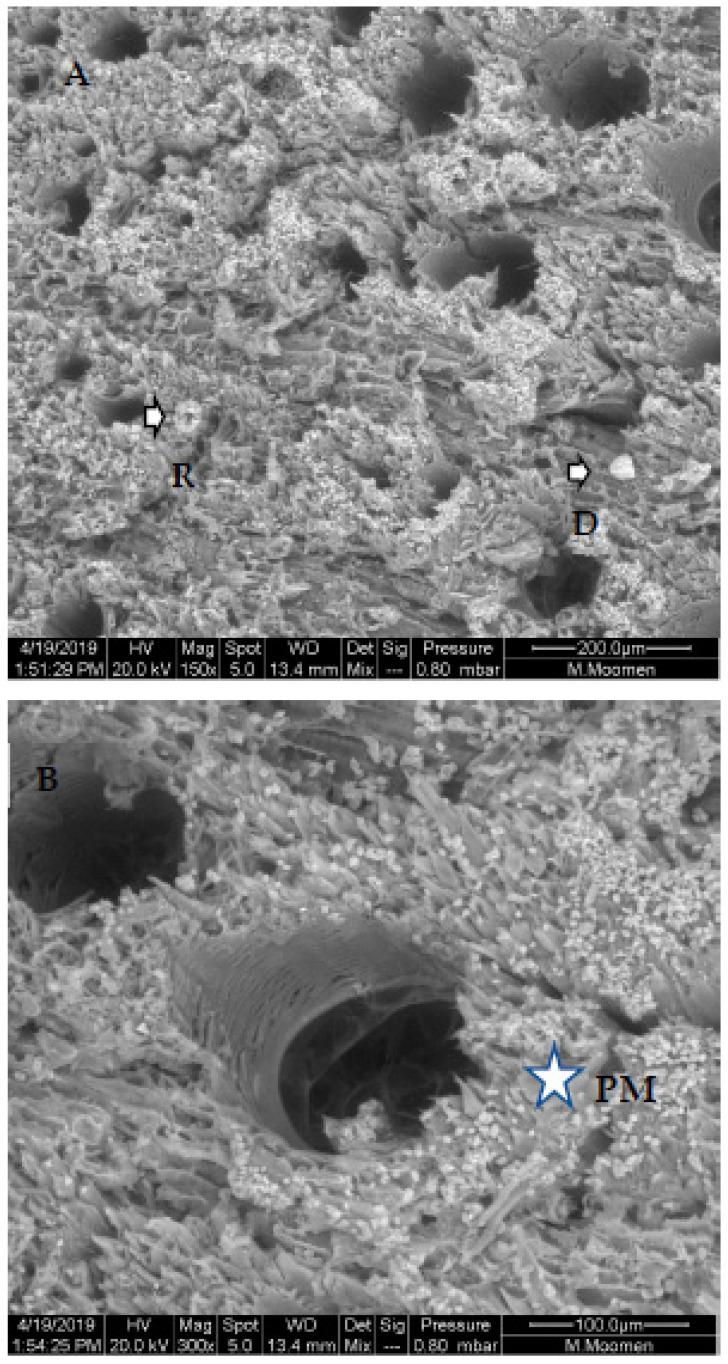
Diversities in crystal morphology and distribution in stem cross section of Sakasly cultivar. (**A**): abundance of crystals visualized on vascular system surface. Note the presence of numerous and rare crystal shape agglomerate in a ring-like shape (R) (arrow) and a drop like shape crystal (D) (arrow). (**B**): an enlarged view of a stem cross section which reveals the detail of an intact pit perforation or pit membrane (PM) marked with an asterix and the abundance of crystals around vessel elements (most of the mineral content is prismatic and spherical crystals).

The examination of xylem vessels using ESEM also revealed the presence of various types of occlusions. Our analysis was focused on the Sakasly cultivar, where the sap flow was higher. The micrograph confirmed the movement of sap flow through intact vessels, pit membranes from which water and solutes circulate (Figure 4). In some cases, a rare crystals shape (drop or ring-like) was observed (Figure 4A). In addition, xylem vessels showed several kinds of crystals, which were potentially deposited along the xylem sap path (Figure 4B).

**Figure 5:**
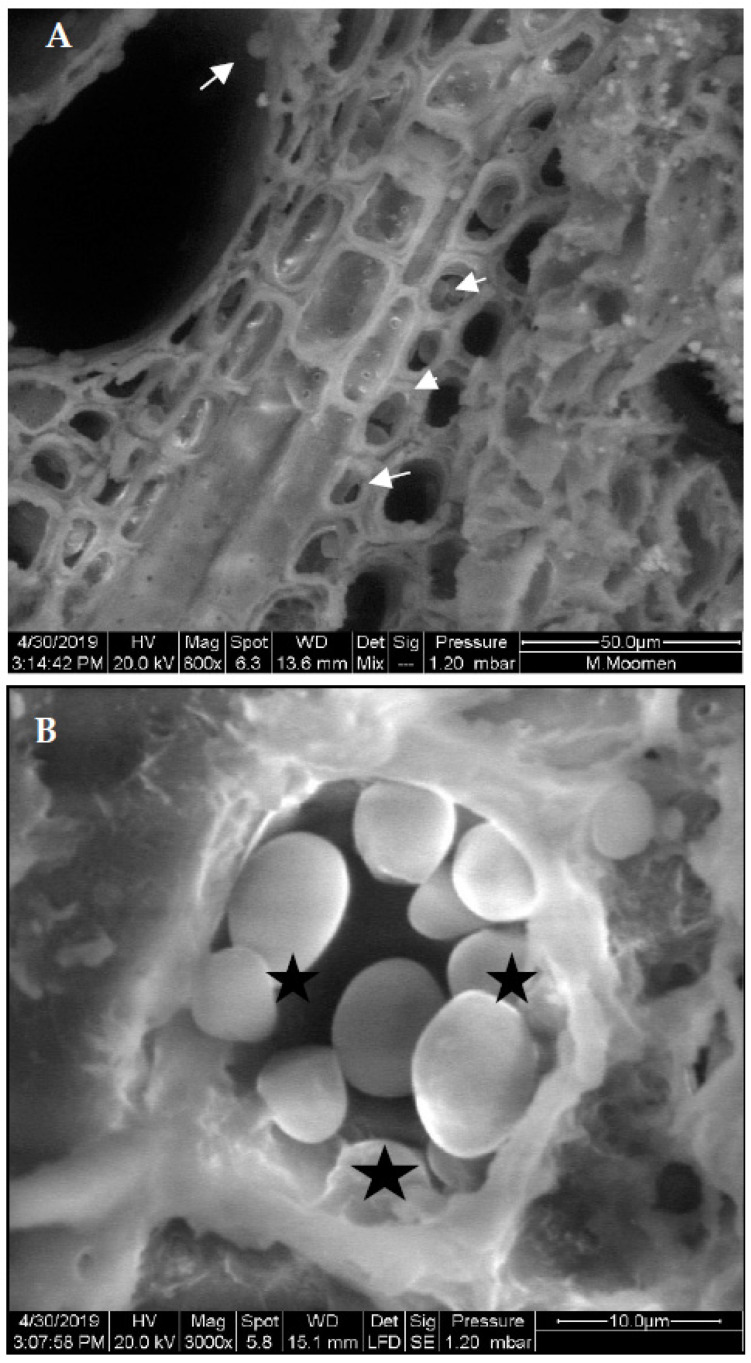
Stem cross section of Razegui1 cultivar imaged in ESEM analysis: (**A**): inserted tyloses in the cell wall of the vessel lumen (white arrow) and in the parenchyma zone (white arrows). (**B**): tyloses visualized at different stage of development inserted in the pit membrane and obstructing the vessel lumen (black asterisks).

**Figure 6:**
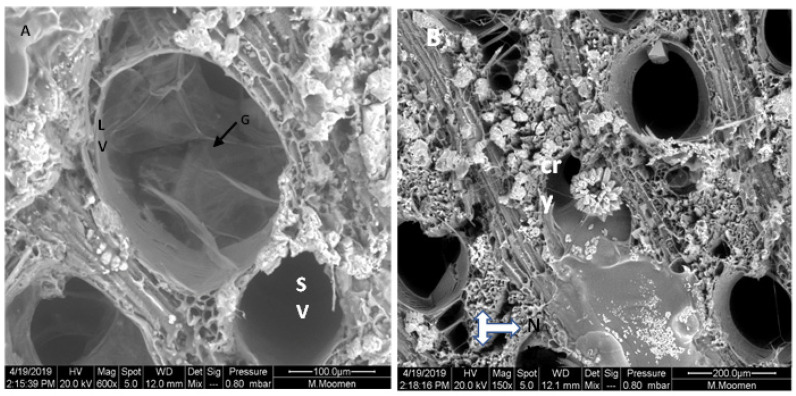
ESEM micrographs of Razegui2 stem cross section revealing in (**A**): an occluded lumen with gel (G) of the large- (LV). and a clear lumen of the small diameter vessels (SV). Note that vessels were surrounded by several crystal units (**B**): in Razegui1, a serial of narrower vessels (NV) do not display any occlusion form in the lumen tissue. Note the presence of crystal units (cry).

Xylem morphology of the different cultivars was marked by the development of tyloses. Their presence in the lumen is in the internal parenchyma of the vessels (Figure 5A), or tyloses can block completely the vessel conducts (Figure 5B). In another case, the stem cross section of Razegui2 revealed that the lumen of the large-diameter (LV) vessels was coated with gel. Meanwhile, the small-diameter vessel presents clear lumen vessels, which were also surrounded by crystal units (Figure 6A). In Razegui1, crystal units presented close to the LV and to the network of narrower vessels (Figure 6B).

**Figure 7:**
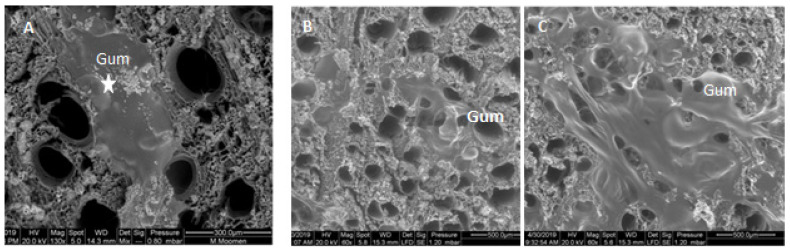
Scanning electron micrographs showing gum occlusions on xylem vessel surfaces. (**A**): entirely obstructed area in Sakasly vessel with gum (asterisks) (**B**,**C**): diffuse and condensed gum deposits on vessel lumens of Khamri and Hencha cultivars.

Gum occlusions were also found in xylem vessel elements, obstructing entirely several vessels (Figure 7A). Diffuse and condensed gum deposits were also identified on Khamri and Hencha cross sections (Figure 7B,C).

**Figure 8:**
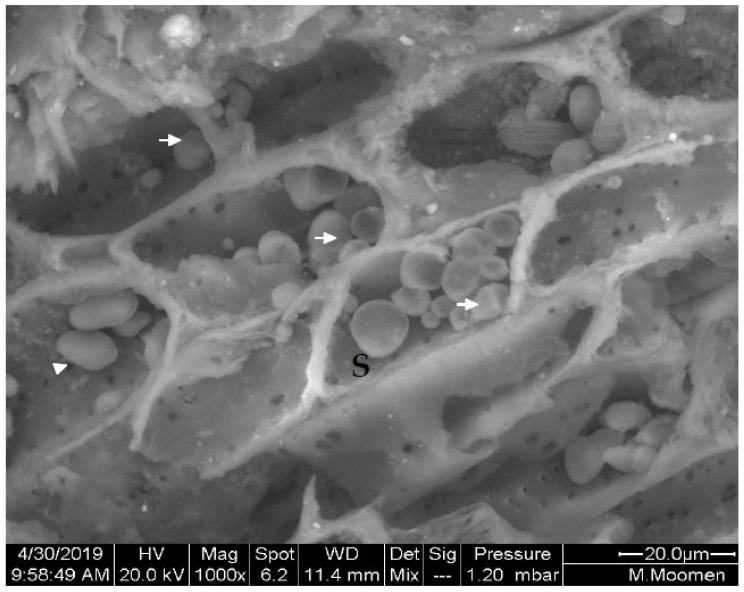
ESEM micrograph of stem cross section in Sakasly cultivar revealing deposition and a dense accumulation of starch grains (S) (white arrows) in parenchymatic pith (A).

**Figure 9:**
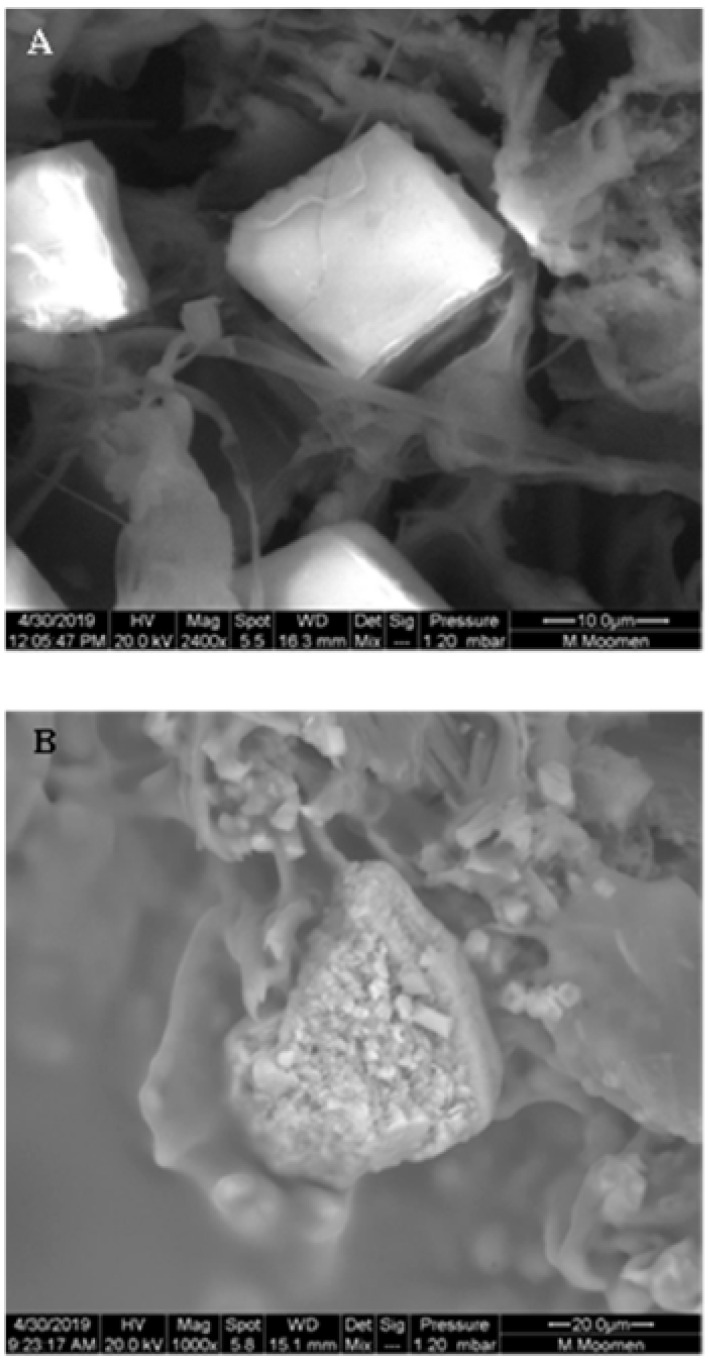
Spatial distribution and diversity of crystal-like shapes visualized by ESEM analysis in a stem cross section. (**A**): Cubic-like shape crystal associated with parenchymatic cell of the vascular system. (**B**): Crystalline pocket containing a cluster of crystalline sand, styloids (rectangular columnar with pyramidal ends) and some irregular other form of crystals dispersed in the parenchymatic tissue.

**Figure 10:**
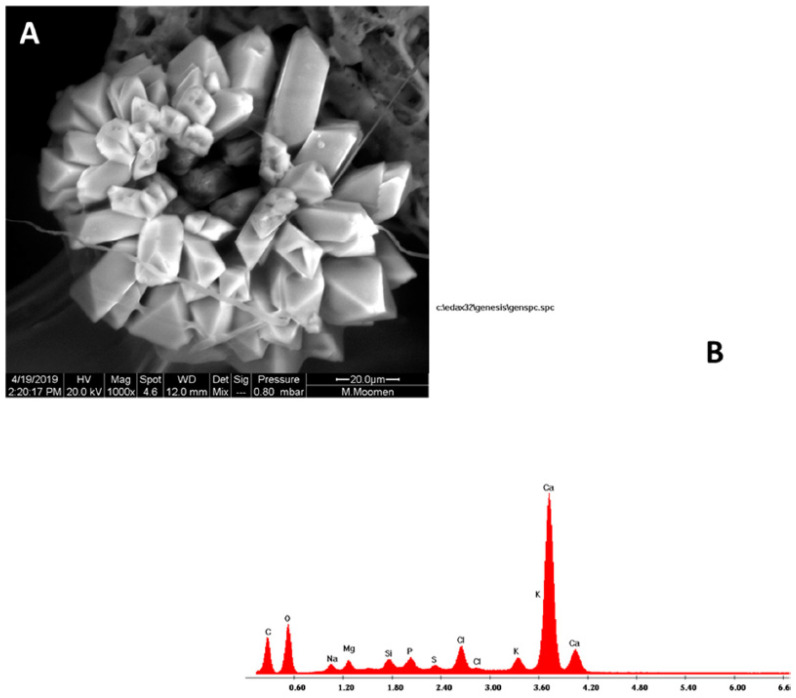
Druse crystal in the vascular system of grapevine visualized by ESEM imaging (**A**): Detail of a dense globular shape with a ring of elements appearing like rose petals or glass flakes. Note the presence of some needle raphide (**B**): X ray showing a spectrum of cells with crystal. The type of crystal is presumably calcium oxalate which showed large Ca, minor C and O peaks, and traces of many other elements.

Vessel elements enriched with starch grains were also detected in stem cross sections. The starch grains exhibited a globular shape with an average diameter of 5 µm (Figure 8). Cross sections also revealed prismatic-like shape crystal formation associated to the parenchymatic tissues of the vascular system (Figure 9A). Crystalline pockets containing a cluster of crystal sand, styloids, and some irregular form of crystals dispersed in the parenchymatic tissue were observed (Figure 9B). Druse crystals crossed by some raphides and a dense globular shape with a ring of elements appearing like rose petals or glass flakes were also visualized (Figure 10A). The X ray analysis (Figure 10B) exhibited a spectrum of crystal, presumably calcium oxalate, with large Ca, and minor C and O peaks. Traces of Mg, Cl, and K were also present in the vessel elements.

An occluded vessel was compared in the stem cross sections of the five cultivars (Table 1). Significant differences among the cultivars were registered regarding the sum of occluded vessels. Sakasly and Razegui1 displayed the highest number of occluded vessel elements, while occlusion sums were not significantly different among Razegui2 (70), Hencha (69) and Khamri (60). The two cultivars Sakasly and Razegui1 presented the sums of 133 and 90, respectively. We also verified that tyloses, gums, as well as starch grains were present in Sakasly.

## 3. Discussion

Unfavourable environmental situations, including drought and vascular pathogens, are responsible for mortality and yield loss in the most vineyards worldwide [24,25]. Viticulturists are interested with grapevine cultivars that provide environmental adaptations and interesting organoleptic characteristics [7,8]. Xylem sap is the first and important physiological response of grapevine after the dormancy period. Results reported in this study, carried out using five local rain-fed grapevine cultivars, supported the difference in sap flow collected during two months of the bleeding sap period. In this study, the xylem sap was examined during three campaigns (2019, 2021, and 2022). The collected sap was accomplished using an easy and simple method. The comparison between the grapevine cultivars during more than one season have not been reported previously. The results showed that the five cultivars have remarkably non-similar xylem sap fluxes. In fact, Sakasly cultivar registered the best sap flow during the 2019 campaign. This result was confirmed during two other consecutive campaigns (2021 and 2022). Sap levels recorded in Razegui1 and Razegui2 are lower than that recorded in Sakasly. In addition, the sap flow of the two clones of Razegui cultivar did not differ significantly from each other, indicating no overall change in the water movement. Khamri and Hencha cultivars appeared to be severely affected by environmental constraints regarding their very low sap flow. Anterior studies confirmed our interest in the xylem sap measurements as a technical method to evaluate water status and water tension inside the xylem [26]. In fact, ref. [26] certifies that xylem sap measurements as well as vessel arrangements are useful criteria to do the distinguishing between vulnerable and tolerant grapevine cultivars towards environmental constraints. We demonstrated that bleeding sap flow is cultivar-dependent, and that the difference in hydraulic function can be under genetic control. This finding was already mentioned in grapevines and in other plant species [11,27,28,29]. Furthermore, our finding was confirmed as the vegetative growth of Khamri and Hencha trees was reduced in comparison with Sakasly, Razegui1 and Razegui2 (data not shown). Similarly, ref. [11] confirmed that xylematic fluxes, in grapevine cultivars, showed variable results. The authors confirmed that the lowest xylem sap translocation in certain cultivar was the recurrent invasion of the cultivars by vascular pathogens. A similar study, based on physiological analysis, including xylem sap measurements and xylem anatomy, revealed that all the tested cultivars (*Persea* species) are susceptible to a lethal vascular wilt disease [30].

Grapevine cultivars, such as Sakasly, Razegui1, and Razegui2, exhibited a better physiological adaptation than Khamri or Hencha. The adaptation consists in a xylem organization able to face external constraints. In accordance with [31,32,33], the authors confirmed, in their study, that xylem rearrangement is extremely important to avoid damages caused by external constraints.

Examination of xylem arrangements through ESEM imaging indicated several scenarios, apparently specific to the cultivars. In fact, completely obstructed areas were observed in Hencha and Khamri, but necrotic lesions as well as sapwood-dwelling pathogens were not identified on our samples. Overall, grapevine genotypes exhibited different responses towards biotic and abiotic stresses and a development of a genotypes-dependant resistance mechanism was even addressed. As demonstrated in our study, water movement in Sakasly, Razegui1, and Razegui2 progressively decreased in consecutive periods (2021 and 2022). However, the total sap flow in those cultivars is higher than the flow registered for Hencha and Khamri. In addition, we demonstrate, in our study, that the sap flow interferes with the existence of a diversified occlusion forms. In agreement with [34,35,36], diversified vascular occlusions are an important tool for plant defence towards wounding injuries or vascular pathogen colonization.

Several types of occlusions were detected in stem vascular system in all the cultivars. Many authors have signaled more than one occlusion form in response to stress [27,37,38]. Additionally, our data confirmed that the number of each form of occlusion was cultivar dependent. However, [35] observed that plants with vascular occlusions produced only one type of occlusion.

Tylose density, size and localization are different within the five cultivars. Those overgrowth entities were present at different developmental stages, ref. [38] related the dynamic process of tylose formation to a wounding repair program or in response to vascular pathogen invasion. Ref. [39] reported that starch reserves were highly represented in resistant grapevine cultivars. This could confirm the ability of Tunisian cultivars to face biotic and abiotic constraints. Additionally, the greater sums of starch grains were registered within the best sap flow cultivars, similarly to [40], who reported large amounts of starch grains in leaves derived from non-infected canes is considered as a first defence step.

Differences in the shape, size and number of intra-lumenal crystals were detected in the vascular system (prismatic, druse styloids and crystalline sand). In their work, ref. [19] confirmed also the existence of three crystal types (druse, prismatic and raphide). Meanwhile, ref. [41] identified raphides or needle-shaped crystals and druses. In our study, an isodiametric and agglomerate druse form appeared as a globular shape and the whole elements appeared as rose are petals or glass flakes in the vascular system. Styloid form detected in the present study was previously described as rectangular columnar with pyramidal ends [42]. Ref. [43] mentioned that high calcium amounts influence both the size and number of druse crystals in the parenchyma of the cells. The existence of such crystal form can be attributed to environment-induced stresses including intracellular regulation of pH, calcium ions and plant defence mechanism. The X-ray microanalysis carried out on our local cultivars showed high concentrations of calcium. Such results were previously reported by [19], who confirmed the existence of calcium-containing crystals in the xylem vessels, but associated to the unexpected presence of pathologies such as *Xylella fastidiosa*. It was also reported that the visualized crystals are most likely calcium oxalate, knowing that it is the most widespread mineral in plant species [19,41,44]. The water bulk that circulates through xylem vessels is responsible for the crystal deposition and can also be the ultimate factor in the modification for the crystal forms found in this study.

According to sap flow and wood anatomy, the five grapevine cultivars presented different degree of tolerance to both biotic and abiotic stresses. In fact, grapevine as a perennial plant was not able to be completely resistant to vascular pathogens [45,46] and/or physical constraints [47,48]. Overall, Sakasly cultivar can be considered as extremely tolerant to such vascular disorders. Razegui1 and Razegui2 may be classified as relatively tolerant to Tunis, 2 July 2022 abiotic constraints. Meanwhile, both Hencha and Khamri cultivars are relatively susceptible.

To the best of our knowledge, this is the first work intended to characterize Tunisian grapevine xylem morphology in relation with xylem sap flow. The different grapevine cultivars studied were classified regarding their sensitivity towards external constraints according to sap flow and xylem morphology. The non-existence of either bacterial or fungal contamination in analyzed grapevine cultivars was confirmed. Diversified occlusion forms have been pointed out in the vascular system of the various cultivars, showing the acquisition of a degree of tolerance of the grapevine, facing external constraints, through a repair process. Additionally, more than five calcium-oxalate shapes were identified in the vessel organs. Among them, several morphological forms were detected for the first time in the local grapevine cultivars, including crystalline pockets as well as ring-; drop-, cubic- like-shapes. The diversity in the sap flow, vascular occlusions, and crystal forms may be attributed to genetic and environmental factors. Although our results are preliminary, physiological and molecular parameters will be particularly useful to discern vulnerability and tolerance between grapevine cultivars.

## 4. Materials and Methods

### 4.1. Plant Material and Sampling

The present study was carried out at an experimental field belonging to the Center of Biotechnology of Borj-Cédria located in the North of Tunisia (36°42′27″ N, 10°25′34″ E). The vineyard was implemented in 1991 and is composed by 61 different cultivars. The genetic relationships of the autochthonous grapevine collection originating from several localities were determined using microsatellites markers [49].

In the present study, Sakasly, Hencha, Khamri, Razegui1, and Razegui2 cultivars were selected according to their interesting organoleptic characteristics [50,51]. Sakasly is one of the most appreciate autochthones cultivars in Tunisia, presenting small bunches, fine-skinned and medium-sized berries. Razegui is cultivated on small areas and is used almost exclusively for the table consumption. The berries are big and white in color. Hencha and Khamri are native grapevine cultivars originated from the south region of the country [52]. Hencha presents medium bunches, fine-skinned and medium-sized white berries [53], while Khamri presents medium-sized red berries. The five genotypes were also selected based on the vine architecture: Sakasly, Razegui1, and Razegui2 are vigorous, while Hencha and Khamri present lower vegetative vigor, reflected in differences in shoot length, density, and leaf color. Razegui is represented by two clones, Razegui1 and Razegui2, and each one is considered as an independent genotype.

The five cultivars were grown under a semi-arid climate and cultivated with the same agronomic practices. The mean annual precipitation at Borj-Cédria is 450 mm, the relative humidity is about 56–73%, and the average annual temperature is 18.7 °C, ranging from 8 °C to 32.9 °C. In the driest months (July and August), a drip irrigation system is managed to overcome water deficit in the vineyard of Borj-Cédria locality. Drip irrigation operates, in most cases, one hour per day, and drip emitters are spaced every 0.30 m in the vine row. The restart of the grape production is accomplished in December of every year by hand pruning, and the experimental vineyard is managed organically with no chemical fertilizer applied. The vines were spaced 2.5 m between rows and 2 m within the row and cultivated according to the double-T training system.

The xylem bleeding sap of the five grapevine cultivars were collected during the natural bleeding period (mid-February 2019, 2021, 2022–mid-April 2019, 2021, 2022) to compare their general fluxes. To improve sap flow, pruning is required (from one year-old fruiting canes) and it is recommended to leave 6–7 buds from the bottom of the fruiting canes. It is also important to leave a portion of wooded cane with 2 cm of distance after the last bud to ensure the tissue repair. For the bleeding sap collection, the extremities of the pruned fruiting canes were inserted in sterile amber bottles and sealed with parafilm for 17 h per day for all samples. At the end of collection, the bottles were tightly closed and stored at 4 °C to be assayed subsequently.

### 4.2. Scanning Electron Microscopy

The extremities of the pruned fruity canes were freshly collected from the five grapevine cultivars in the spring (April), just before budbreak. On fruiting canes with 6 to 7 buds, the cross sections were made at 2 cm after the extreme bud, using sterile razor blades. Samples slices of 100 μm thick were picked up onto carbon double-faced sticky tape mounted onto aluminium stubs. The sections were inspected using an Environmental Scanning Electron Microscope (ESEM). The standard operating conditions of the ESEM were adjusted in the range of 5 and 20 kV to optimize the observations. Energy dispersive X-ray analysis (EDX) was conducted using a detector with a QDD Violin Detector (8-μm beryllium window) and VIDXScan Active Digital Imaging software.

### 4.3. Vessel Occlusion Analysis and Mineral Composition

To identify and specify the degree and the nature of the samples obstructions and the nature of mineral composition, stem cross sections were analyzed using an ESEM system. During pruning time and for each cultivar, dormant canes were sectioned for analysis into 20–30 μm sections with a vertical microtome (Reichert, Wien, Austria). To avoid artificial gum secretion, stem cross sections were analyzed freshly and without prior chemical fixation. For each cross section, only the quarter of the section was examined.

## Figures and Tables

**Table 1:** Sum and type of occlusions recorded in xylem vessels among stem cross sections of local grapevine cultivars.

Cultivars	Tyloses	Gums	Gels	Starch Grains	Occlusion Sums
Sakasly	25	33	0	75	133
Razegui1	13	22	0	55	90
Razegui2	11	9	11	39	70
Hencha	21	6	21	21	69
Khamri	27	5	17	11	60

## Data Availability

The data generated and analyzed during this study are included in this article.
